# Identification of a stem cell mediating osteoblast versus adipocyte lineage selection

**DOI:** 10.21203/rs.3.rs-198922/v1

**Published:** 2023-01-26

**Authors:** Matthew Greenblatt, Shawon Debnath, Alisha Yallowitz, Jason McCormick, Sarfaraz Lalani, Tuo Zhang, Michelle Cung, Seoyeon Bok, Jun Sun, Hiranmayi Ravichandran, Yifang Liu, John Healey, Paul Cohen

**Affiliations:** Department of Pathology and Laboratory Medicine Weill Cornell Medicine; Weill Cornell Medicine, Cornell University; Cornell University; Weill Cornell Medicine, Cornell University; Weill Cornell Medicine, Cornell University; Weill Cornell Medical College; Weill Cornell Medicine, Cornell University; Weill Cornell Medicine, Cornell University; Weill Cornell Medicine, Cornell University; Weill Cornell Medicine; Immunopathology Laboratory, New York Presbyterian; Memorial Sloan Kettering Cancer Center; The Rockefeller University

**Keywords:** Stem cell, Bone, Adipocytes, Osteoblasts

## Abstract

Most skeletal fragility disorders are characterized by bone loss with a concurrent gain in marrow adipocytes 1–8. This suggests that a cell that forms adipocytes at the expense of osteoblasts is central to the pathogenesis of skeletal disorders. However, this cellular point of bifurcation between adipocyte and osteoblast differentiation pathways remains unknown. Here, we identify a new cell type defined by co-expression of skeletal stem cell and adipocyte precursor markers, 9–13 (CD24+CD29+ skeletal stem cells (SSCs)), that serves as a key cellular point of bifurcation between the osteoblast and adipocyte differentiation pathways, giving rise to closely related osteoblast and adipocyte lineage-restricted precursors. CD24+CD29+SSCs comprise a small fraction of SSCs, and only this fraction displays full stemness features, including the ability to undergo serial transplantation. In line with serving as the osteoblast/adipocyte bipotent cell, the “bone to fat” tissue remodeling occurring in models of postmenopausal osteoporosis or after high fat diet exposure occur in part by reprogramming these CD24+CD29+SSCs to change their output of lineage-restricted precursors. Lastly, as subcutaneous white adipose tissue displays a similar set of CD24+CD29+ stem cells and related lineage-restricted progenitors, these findings provide a new schema explaining the stem cell basis of bone versus adipose tissue production that unifies multiple mesenchymal tissues.

The bifurcation of differentiating skeletal progenitors into osteogenic and adipogenic lineages is a critical cellular decision point that is a key determinant of skeletal tissue composition. To gain insight into this point of bifurcation, we tracked a specific population of periosteal stem cells (PSCs) ^10^ that, in contrast with marrow resident endosteal cells, only gains adipogenic capacity post-fracture, allowing real-time observation of early lineage commitment and subsequent differentiation into the osteogenic versus adipogenic cellular sequence (Fig 1 a–b, Extended Data Fig 1 a-b). The cells within this PSC lineage defined by a CTSK-cre activated mGFP reporter were analyzed in mice by flow cytometry with a combination of markers used to identify SSCs ^9,10^ ( Lin-THY-6C3-CD200+CD105-) and adipocyte precursors ^11,13^ (CD24 and CD29). Fracture rapidly induced >10-fold expansion in a novel population expressing both adipose progenitor-associated CD24 and CD29 and SSC markers (CD24+CD29+SSCs) (Fig 1b)^10–12^. Additionally, a large population of CD29+SSCs was present during the early healing phase of active osteogenesis. Later, with kinetics matching the timing of adipocyte production, a CD24+SSC population emerged, showing a >40-fold enrichment relative to the contralateral leg (Fig 1b, Extended Data Fig 1 a). Many populations outside of the SSC definition, such as CD105+ cells, could be excluded as the point of osteoblast/adipocyte bifurcation, as these are labeled by *Fabp4*^*cre*^, which labels committed adipocyte lineage cells (Extended Data Fig 1c)^14^. *Pdgfrn*^*cre*^, which labels both osteoblasts and adipocytes and therefore, also likely labels a pre-lineage commitment bipotent population, showed robust labeling of CD24+CD29+SSCs (Extended Data Fig 1d)^13–17^. Based on these observations, candidates were nominated for the bipotent and osteoblast/adipocyte restricted cell types: CD24+CD29+SSCs as a bipotent *bona fide* stem cell able to generate both osteoblasts and adipocytes in bone, CD24+SSCs as adipocyte-restricted progenitors, and CD29+SSCs as osteoblast-restricted progenitors (Fig 1c).

Developmental analysis corroborates this schema and indicates that it applies across multiple mesenchymal tissue types, including the marrow resident SSCs outside the periosteum (Fig 1d). In the early postnatal period, bone solely displays osteogenic output, only gaining the capacity to produce mature marrow adipocytes at approximately 4–6 weeks of age in mice. Thus, the first skeletal adipocytes developmentally predate other populations previously considered as marrow adipocyte precursors, such as LEPR+ Cxcl12 abundant reticular (CAR) cells ^18–21^. Similarly, cre lines labeling CAR cells only provide partial labeling of the skeletal adipocyte pool under many conditions. Thus, while CAR cells can contribute to marrow adipogenesis, such as with aging or irradiation, they cannot account for all marrow adipogenesis. In this respect, it is notable that CD24+CD29+SSCs do not overlap with LEPR+ cells (Extended Data Fig 1e). We observed that in the early postnatal period that bone solely contains CD24+CD29+ and CD29+ SSCs, whereas CD24+SSCs emerge at approximately 6 weeks of age with a timing matching adipogenesis (Fig 1 d–e, Extended Data Fig 1b, 1 f-g). Additionally, to see if this schema may apply to multiple mesenchymal tissues, subcutaneous white adipose tissue (sWAT) was similarly examined, finding that it also contained CD24+CD29+SSCs and abundant putatively adipogenic CD24+SSCs expressing the adipocyte lineage marker CD36 while lacking putatively osteogenic CD29+SSCs (Fig 1f, Extended Data Fig 1 h-i) ^22^. Similar findings were observed in intrascapular and visceral adipose tissue (Extended Data Fig 1j-k).

*In vivo* tissue production assays were then used to directly establish the osteoblast/adipocyte bipotency of CD24+CD29+SSCs. Only CD24+CD29+SSCs and CD29+SSCs displayed intrinsic osteogenic capacity through the ability to form bone organoids *in vivo* after isolation and transplantation into the kidney capsule of secondary hosts (Fig 2a–b). Conversely, only CD24+CD29+SSCs and CD24+SSCs displayed adipogenic capacity in the same system, staining positive for LipidTOX, with CD24+CD29+SSCs generating bone organoids encapsulating a marrow-like space including Perilipin1+ adipocytes (Fig 2c–d). CD24−CD29−SSCs displayed neither robust osteogenic nor adipogenic capacity.

Similarly, only CD24+SSCs and CD24+CD29+SSCs or their counterparts with identical surface immunophenotypes from sWAT were able to reconstitute adipogenesis when transplanted into the residual mammary fat pad of “fatless” *A-Zip/F1* hosts ^23^. Use of “fatless” *A-Zip/F1* hosts allows for both assessment of adipogenesis and functional assessment of adipocyte reconstitution via rescue of the severe lipoatrophy of these hosts without the presence of confounding host adipocytes ^11^. Only CD24+CD29+SSCs and CD24+SSCs and their sWAT counterparts were able to reconstitute adipogenesis after transplantation (Fig 2e–j, Extended Data Fig 2a-c). Not only did these two populations from either bone or fat sources physically reconstitute adipocytes at the gross, histologic and immunophenotypic levels, but they functionally reconstituted adipogenesis as seen through suppression of the diabetic phenotype associated with the severe lipoatrophy in *A-Zip/F1* mice or through the reconstitution of systemic adipokine levels (Fig 2k i and iii, Extended Data Fig 2d i and iii) ^11,23^. In keeping with the model that CD24+CD29+SSCs give rise to adipocyte committed and therefore non-stem CD24+SSCs, analysis of extended timepoints after transplantation showed that only the reconstitution by CD24+CD29+SSCs was durable, whereas the physical and functional reconstitution of adipogenesis by CD24+SSCs had largely abated by 20 weeks post transplantation (Fig 2k ii and iv, 2l, Extended data Fig 2d ii and iv).

This finding that only a small CD24+CD29+ fraction within the current definition of SSCs were capable of durable reconstitution of adipogenesis implied that CD24+CD29+ cells may be the only truly stem fraction of SSCs, thereby prompting a revision of the SSC definition. To test this directly, each of the CD24/CD29-defined fractions of SSCs was subjected to transplantation-based assessment of self-renewal and differentiation hierarchy (Fig 3a). CD24+CD29+SSCs were capable of self-renewal, being able to maintain their immunophenotype through transplantation (Fig 3d–e). CD24+CD29+SSCs sat at the apex of their differentiation hierarchy, being able to generate CD29+SSCs and more mature CD105+, THY+ and 6C3+ derivatives when transplanted into wild type mammary fat pads (Fig 3d, Extended Data Fig 3a). Interestingly, CD24+CD29+SSCs favored production of CD24+SSCs when transplanted into residual fat depots of *A-Zip/F1* hosts, highlighting how differences in host environment regulate the lineage commitment of this bipotent population (Fig 3e). In contrast, CD29+SSCs did not give rise to the CD24+CD29+SSCs after transplantation (Fig 3f). CD24+SSCs showed only limited engraftment in wild type hosts (Fig 3b) but engrafted and rapidly differentiated into a 6C3+ population without generating other CD24/29-defined SSC subsets when transplanted into *A-Zip/F1* hosts (Fig 3c). CD24−CD29−SSCs failed to engraft after transplantation (Fig 3g). Therefore, CD24+CD29+SSCs are the only fully stem fraction of SSCs, as only CD24+CD29+SSCs self-renew and sit at the apex of their differentiation hierarchy.

Current methods for tracking the differentiation of specific types of skeletal cells rely exclusively on cellular transplantation and subsequent re-isolation of derived populations, which, while a powerful approach, is limited by the tissue disruption associated with the transplantation procedure. To counter this limitation and determine lineage and differentiation relationships among skeletal cell types in their native environment, we modified recently described methods for tracking clonal and sub-clonal mitochondrial mutations across populations. Specifically, we utilized *PolG*^*D257A/D257A*^ mice with a mitochondrial DNA “mutator” phenotype that provide a rich substrate of mutations for analysis ^24,25^.Each of the CD24/CD29-defined SSC populations and other skeletal populations including CD105+, THY+, 6C3+ or THY+6C3+ cells were isolated by FACS from 3-month old *PolG*^*D257A/D257A*^ femurs and were subjected to analysis of mitochondrial DNA mutations by ATAC-seq based mitoDNA capture ^24^. A number of clonal variants were observed to be shared among CD24+CD29+, CD24+ and CD29+ SSCs, which, together with differentiation hierarchy studies, provides direct evidence that bipotency of CD24+CD29+SSCs is observed at the level of single clones (Extended Data Fig 3b-d). A cluster dendrogram was constructed in an unsupervised manner from these variants, placing CD24+CD29+SSCs at the first branch of this dendrogram in line with our differentiation hierarchy model (Fig 3h). When considered alongside transplantation-based differentiation hierarchy studies, CD24+CD29+SSCs clonally transmit variants to each of the other populations isolated, and thus CD24+CD29+SSCs display multipotency for other skeletal cell types in the native bone environment (Fig 3i).

Consistent with our model, analysis of the genomic DNA portion of the ATAC-seq data demonstrated broadly different epigenetic profiles for CD24+CD29+, CD24+ and CD29+ SSCs (Fig 3j, Extended Data Fig 3e). There was decreased accessibility of several genes associated with adipogenesis (*Fasn, Cebpg, Stat5a, Stat6*) accompanying the osteoblast commitment seen in CD29+SSCs. However, the reciprocal effect of silencing of osteoblast-lineage associated genes (*Sp7, Spp1, Crtap*) in CD24+SSCs was less pronounced (Fig 3k).

The physiologic bipotency of CD24+CD29+SSCs for osteoblast and adipocyte generation was confirmed in the native bone environment of irradiated *A-Zip/F1* hosts using orthotopic transplantation directly into the femoral marrow cavity. Indeed, after orthotopic transplantation CD24+CD29+SSCs directly gave rise to both RUNX2+ osteoblasts in trabecular bone and to mature bone marrow adipocytes positive for FABP4 and LipidTOX staining (Fig 4a–d). In addition to these mature pools of cells, CD24+CD29+SSC graft-derived cells also incorporated into the growth plate (Fig 4e), in line with recent reports describing the growth plate region as a potential reservoir for skeletal stem cells ^9,26^. In keeping with this growth plate localization, a portion of the CD24+CD29+SSC derived cells are labeled by a PTHrP-mCherry reporter previously associated with a growth plate resident label retaining multipotent population ^26^ (Fig 4f). Similar to findings in organoid systems, transplanted CD24+SSCs only gave rise to bone marrow resident LipidTOX+ adipocytes (Fig 4g–h), and CD29+SSCs only formed RUNX2+ osteoblasts (Fig 4i–j). Both CD24+ and CD24+CD29+ SSCs provided functional reconstitution of adipogenesis in the orthotopic setting, as shown by significant suppression of the *A-Zip/F1* diabetic phenotype and a significant increase in circulating adiponectin levels (Fig 4k).

Previously, the large number of markers needed to define SSCs and related progenitor populations has precluded determination of their location and, accordingly, the physical trajectory of differentiating skeletal cells. To address this, imaging mass cytometry was performed by applying a 13-color panel that included the flow cytometry markers used to define these populations (Fig 4l–n, Extended Data Fig 4a-r). Robust staining was observed in all regions except the growth plate, which was excluded from further analysis. Segmentation and gating on per cell marker expression were used to localize populations matching the flow cytometry defined cell types studied above (Fig 4o–r, 4w). This revealed that CD24+CD29+SSCs were enriched in the secondary ossification center and the primary spongiosum near the growth plate and included many Nestin+ cells (Fig 4o, 4q, Extended Data Figs 4c-d, 4i, 4m, 4p) ^27^. Consistent with the physiologic absence of adipogenesis in the periosteum, we observed that periosteum lacked CD24+SSCs and only contained osteoblast committed CD29+ SSCs (Fig 4p). To elucidate whether there is a physical organization of differentiating cells derived from CD24+CD29+SSCs that matches their transplantation determined differentiation hierarchy, the cell types that serve as the nearest cellular neighbors of CD24+CD29+SSCs were calculated. In line with our differentiation hierarchy studies (Fig 3), the most likely nearest neighbors for CD24+CD29+SSCs, aside from other CD24+CD29+SSCs, are CD24+SSCs and CD29+SSCs or other CD29+ populations with a similar immunophenotype (Fig 4s–w, Extended Data Fig 4s-v). Thus, the physical proximity of these subsets supports the model of lineage bifurcation and differentiation hierarchy observed in transplantation systems and is consistent with a physical flow of differentiating cells stemming from CD24+CD29+SSCs.

“Bone to fat” tissue remodeling is a signature of multiple disease states, including osteoporosis or obesity. Identifying CD24+CD29+SSCs as the point of adipocyte/osteoblast lineage bifurcation enables deconvolution of the relative contribution of alterations in lineage commitment versus changes in the differentiation/function of mature post-commitment cell types to these disorders. Indeed, in tandem with high fat diet (HFD)-associated positive energy balance (HFD-PEB) causing “bone to fat” remodeling of the skeleton (Extended data Fig 5a-d), the total numbers of adipogenic committed CD24+SSCs were significantly increased with a decrease in osteogenic committed CD29+SSCs (Fig 5a–b, Extended Data Fig 5e). To determine the cellular basis of how HFD-PEB acted on CD24/CD29-defined SSC subsets to produce this “bone to fat” remodeling, each of these subsets were isolated and transplanted into hosts exposed to matching high fat or control low fat diets for 12 weeks (Fig 5c). HFD-PEB acted directly on transplanted CD24+CD29+SSCs to both decrease their osteogenic output and increase their adipogenic output (Fig 5d–f, Extended Data Fig 5f-g). Thus, HFD-PEB acts directly on CD24+CD29+SSCs to change their characteristic output of osteoblast versus adipocyte committed progenitors as a means to effect tissue remodeling. HFD-PEB also acted on transplanted lineage committed CD29+SSCs to decrease their bone formation capacity but did not introduce post-commitment lineage plasticity as CD29+SSCs did not display a gain in adipogenic capacity (Fig 5d–f). Consistent with HFD-PEB directly reprogramming CD24+CD29+SSCs, broad transcriptional changes in CD24+CD29+SSCs were observed after HFD, including upregulation of *Bmp1, Bmp5* and adipogenesis associated genes (*Adam8, Gas6, Foxc1*) ^5,28,29^ while expression of genes associated with stemness or specifically with SSCs was maintained (*Bmp2, Klf4, Sox9, Hes1, Hey1*) ^9,10,30^ (Fig 5g–h, Extended Data Fig 5h).

Next, we assessed whether direct reprogramming of CD24+CD29+SSCs is a convergent mechanism for tissue remodeling occurring in multiple skeletal disease states, including postmenopausal osteoporosis. Indeed, mice undergoing the ovariectomy model of post-menopausal osteoporosis displayed a significant expansion in adipogenic committed CD24+SSCs and contraction of osteogenic committed CD29+SSCs similar to that observed after HFD exposure (Fig 5i–j, Extended Data Fig 6a-g). This, likewise occurred due to ovariectomy directly reprogramming CD24+CD29+SSCs to skew these cells away from the production of osteoblast committed and towards adipocyte committed progenitors, as seen with CD24+CD29+SSCs isolated from unmanipulated donors and transplanted into overiectomized hosts (Fig 5k–n, Extended data Fig 6h-k). This enhanced adipocyte and suppressed osteoblast lineage commitment by CD24+CD29+SSCs was durable and cell intrinsic, as seen through transplantation of CD24+CD29+SSCs from overiectomized donors into non-overiectomized hosts (Extended data Fig 6l-o). These findings are consistent with the decreases in osteoblast numbers and bone formation and concurrent increases in marrow adipogenesis seen at late timepoints either post ovariectomy in mice or in human postmenopausal osteoporosis (Extended data Fig 6j-k) ^4,8,31^. Thus, both ovariectomy and HFD-PEB act directly on CD24+CD29+SSCs to skew their production of lineage committed progenitors during the bifurcation step.

Taken together, we here identify that CD24+CD29+SSCs represent a key point of bifurcation between lineage committed CD24+SSC adipocyte precursors and CD29+SSC osteoblast precursors and the only fully stem fraction of SSCs. Skeletal disorders act directly on CD24+CD29+SSCs to increase their adipogenic commitment and decrease their osteogenic commitment, indicating a direct competition for lineage allocation in these cells in at least a subset of bone disorders. Insofar as these cells and their functions are preserved across both bone and sWAT tissue, this represents the first example of a cross-tissue schema identifying a discrete, physiologic stem cell identity shared across multiple mesenchymal tissue types.

## Figures and Tables

**Figure 1:**
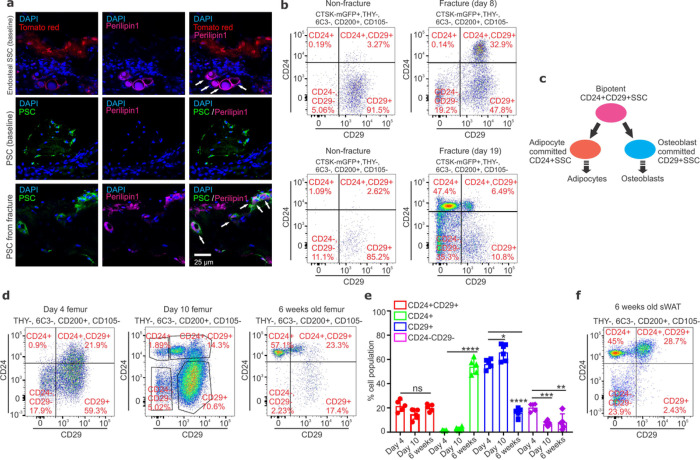
CD24 and CD29 define discrete SSC subsets. **a,** Endosteal SSCs (tomato red) isolated from non-fractured femurs (baseline, top) and CTSK-mGFP+ periosteal stem cells (PSCs, green) isolated from non-fractured (baseline, middle) or fractured femurs (bottom) were transplanted into the kidney capsule of *MIP-GFP* recipient mice. 6 weeks later, immunostaining for Perilipin 1 (magenta; white arrows indicate co-localization) and DAPI (blue) for nuclei was performed. Scale bar 25μm. **b,** FACS plots showing PSCs isolated from non-fractured (left column) and fractured (right column) femurs 8 (top) and 19 days (bottom) after fracture. **c,** Proposed model for osteoblast versus adipocyte lineage commitment. **d,** Representative FACS plots showing the distribution of skeletal stem cell (SSC) subsets isolated from femurs of 4 day (left), 10 day (middle), and 6 week (right) old mice. A detailed strategy for FACS isolation of SSC subsets is provided in Extended Data Fig 1b. **e,** % of CD24+CD29+, CD24+, CD29+ and CD24− CD29− SSC populations in long bones at the indicated ages. Significant increase in CD24+SSCs at 6 weeks (****p <0.0001); significant increase (*p =0.0243) at day 10 and significant decrease (****p <0.0001) at 6 weeks in CD29+SSCs; significant decrease in CD24−CD29−SSCs at day 10 (***p = 0.0002) and 6 weeks (**p =0.0026). One-way ANOVA, Tukey’s multiple comparison test; mean ± S.D; n=5 to 6, ns= non-significant. **f,** FACS plot showing the distribution of CD200+ mesenchymal cells in murine subcutaneous white adipose tissue (sWAT) at 6 weeks of age.

**Figure 2:**
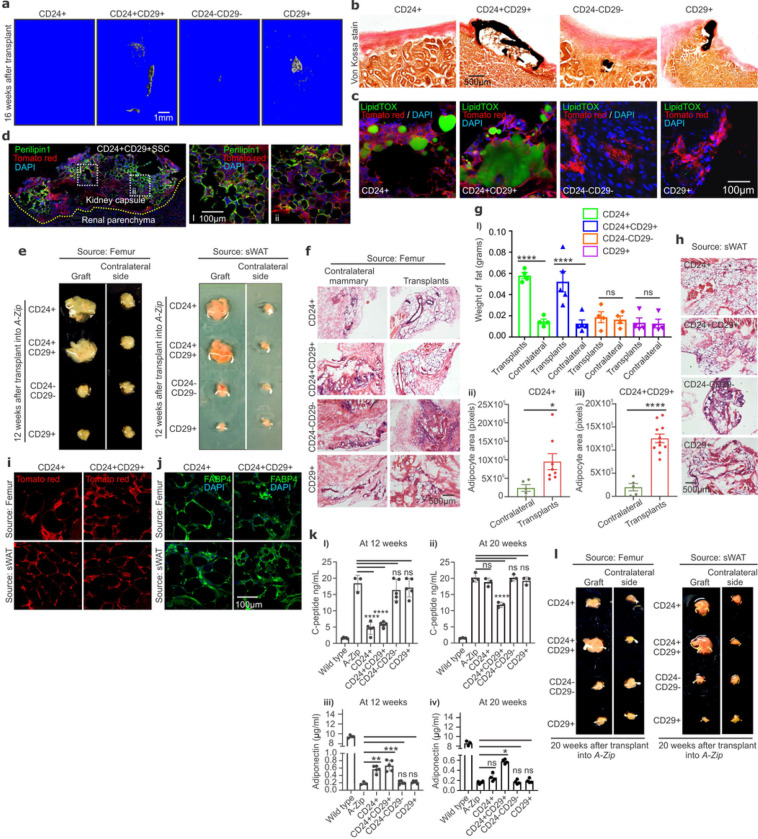
CD24 and CD29 define bipotent and lineage committed SSC subsets. **a-b,** CD24+, CD24+CD29+, CD24−CD29− and CD29+ SSCs (left to right) were transplanted into the kidney capsule of *MIP-GFP*recipients, and bone organoids formed after 16 weeks were visualized by μCT **(a)** and von Kossa staining (black) **(b).** Scale bar 1mm (a) and 500μm (b). **c,** LipidTOX staining (green) confirms adipogenesis by the transplanted cells (tomato red). DAPI (blue) for nuclei. Scale bar 100μm. **d,** CD24+CD29+SSCs (tomato red) were allowed to form organoids after transplantation, and graft derived adipogenesis (Perilipin 1 staining, green) is shown. Scale bar 100μm. Enlarged view of dotted white boxes (i-ii). DAPI (blue) for nuclei. **e,** CD24+, CD24+CD29+, CD24−CD29− and CD29+ SSCs (top to bottom) from mouse femurs (left) or subcutaneous white adipose tissue (sWAT, right) were transplanted into *A-Zip/MIP-GFP* hosts. Reconstitution of adipose tissue by the graft compared to the contralateral residual adipose depots, representative gross images taken 12 weeks post-transplantation. **f,** H and E staining of the tissues in (e), derived from femoral skeletal cells. Scale bar 500μm. **g, (i)**, Weight of reconstituted graft (transplants) adipose tissue derived from femoral skeletal cells in (e). Significant increase in adipose tissue produced by CD24+ (****p <0.0001) and CD24+CD29+ (****p <0.0001) SSCs. One-way ANOVA, Sidak’s multiple comparison test; mean ± SEM; n=4 to 5, ns= non-significant. **ii-iii**, Transplanted CD24+ **(ii)** and CD24+CD29+ **(iii)** SSCs show a significant increase (*p =0.0301; ****p<0.0001) in areal measures of adipocyte formation. Mean ± SEM; n=5 to 8; 2 tailed Student’s t-test. **h,**H and E staining of graft tissue derived from sWAT as shown in (e). **i-j,**Reconstituted adipose tissue from CD24+ (left), and CD24+CD29+ (right) SSCs isolated from femurs (top) or sWAT (bottom) is donor derived as confirmed by mTomato (red) expression **(i),** and staining for Fabp4 (green) **(j)**. DAPI (blue) for nuclei. Scale bar 100μm. **k,** Functional reconstitution of adipose tissue by CD24/CD29 SSC subsets isolated from femurs and transplanted into *A-Zip/MIP-GFP* hosts as shown by plasma levels for Insulin C-peptide (top), and adiponectin (bottom) at 12 **(i, iii)** and 20 weeks **(ii, iv)**. Significant decrease in C-peptide by transplanted CD24+ (****p < 0.0001) and CD24+CD29+ (****p <0.0001) SSCs **(i),** and a significant increase in plasma adiponectin by CD24+ (**p =0.0057) and CD24+CD29+ (***p = 0.0005) SSCs **(iii)** at 12 weeks. Significant decrease in C-peptide (****p <0.0001) **(ii)**, and significant increase in plasma adiponectin (*p =0.0315) by CD24+CD29+SSCs **(iv)** at 20 weeks. One-way ANOVA, Tukey’s multiple comparison test; mean ± S.D; results representative of 3 to 5 independent experiment, ns= non-significant. **l,** Transplantation of CD24+, CD24+CD29+, CD24−CD29− and CD29+ SSCs (top to bottom) isolated from mouse femurs (left) or sWAT(right) into *A-Zip/MIP-GFP* hosts, demonstrating reconstitution of adipose tissue vs the host contralateral side after 20 weeks.

**Figure 3:**
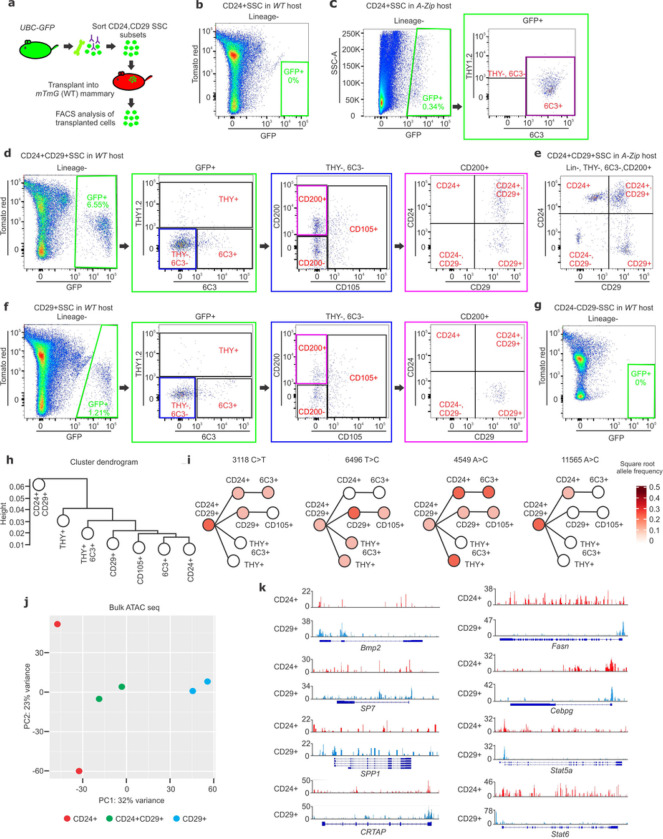
Differentiation hierarchy of the CD24 and CD29 defined SSC subsets. **a**, Schematic representation of transplantation of sorted skeletal stem cell (SSC) subsets into mouse mammary fat pad for 2 weeks. **b-c,** FACS plots for CD24+SSCs after the first round of transplantation into mammary fat pad of WT (b) or *A-Zip/MIP-GFP* hosts **(c). d-e,** FACS plots for CD24+CD29+SSCs after first round of transplantation into the mammary fat pad of WT **(d)** or *A-zip/MIP* hosts **(e). f-g,** FACS plots for CD29+SSCs **(f),** and CD24−CD29−SSCs **(g)** after the first round of transplantation into the mammary fat pad of WT hosts. (Lineage- = Ter119−, CD45− and CD31−). Color coded boxes (green, blue and magenta) indicate parent/ daughter gates. **h,** A dendrogram was obtained through unsupervised hierarchical clustering of the high confidence mitochondrial DNA mutations in different skeletal cell populations (refer to Extended Data Fig 3b-d). **i,** Allele frequency overlaid on a transplantation determined model of cellular differentiation. Allelic heteroplasmy of four selected variants showing propagation of the mitochondrial mutations among skeletal cell subsets. **j,** Principal component analysis of ATAC sequencing performed on sorted CD24+, CD24+CD29+ and CD29+ SSCs isolated from 3-month-old *PolG*^*D257A/D257A*^ mouse femurs. **k,** ATAC-determined chromatin accessibility peaks are shown for *Bmp2, SP7, SPP1, CRTAP* (left) and *Fasn, Cebpg, Stat5a, Stat6* (right) comparing CD24+ and CD29+ SSC populations.

**Figure 4:**
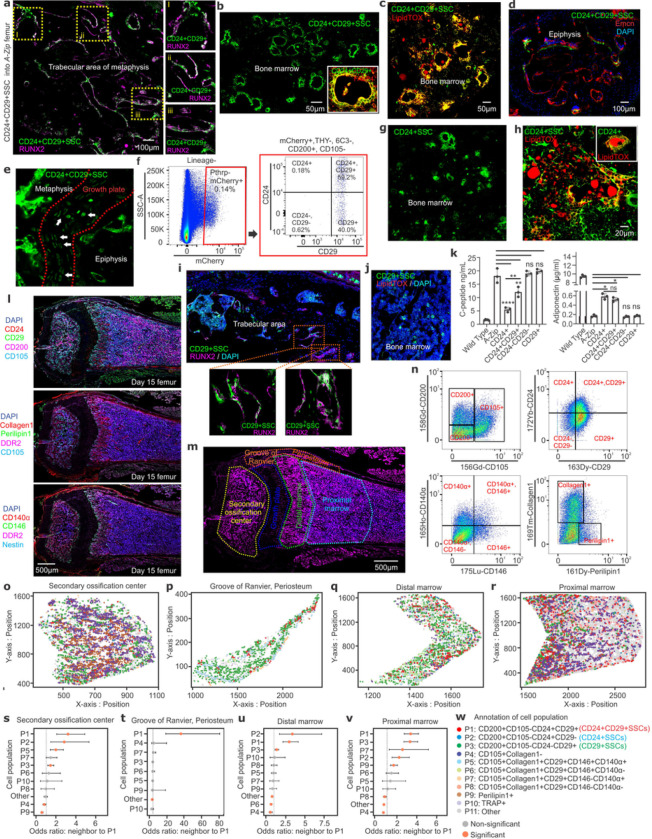
The location of CD24 and CD29 defined SSC subsets in bone. **a-e,** Orthotopic transplantation of CD24+CD29+SSCs (green) into the femoral cavity of irradiated *A-Zip/ MIP-GFP* mice for 12 weeks. Images represent engrafted transplanted cells in host trabecular bone including osteoblast differentiation of graft cells as shown by RUNX2 (magenta) staining. Enlarged images for dotted yellow boxes (i-iii) **(a)**. Orthotopically transplanted CD24+CD29+SSCs (green) engrafted in host bone marrow, differentiated into adipocytes shown by Fabp4 (red) **(b)** and LipidTox (red) staining **(c),** engrafted in the host epiphysis adjacent to Endomucin+ (red) endothelial cells **(d),** and localized into the host growth plate (white arrows) **(e)**. DAPI for nuclei. Scale bar 100μm (a, d), 50μm (b, c). **f,** FACS plots for Pthrp-mCherry+ cells isolated from the femurs of 15-day old *PTHrP*^*mcherry*/+^ mice. **g-h,** Transplanted CD24+SSCs (green) reconstituted into the bone marrow of secondary hosts **(g),** and differentiated into LipidTOX + (red) adipocytes **(h).** Scale bar 20μm. **i-j,** Transplanted CD29+SSCs (green) engrafted in host trabecular bone, differentiated into osteoblasts as shown by RUNX2 (magenta; enlarged view of dotted orange boxes) staining **(i),** and reconstituted in bone marrow staining negative for LipidTOX (red) **(j)**. DAPI for nuclei. **k,** Intrafemoral transplantation of the indicated cells into *A-Zip* hosts resulted in functional reconstitution of bone marrow adipocytes as measured by Insulin C-peptide suppression (left), and adiponectin reconstitution (right) at 12 weeks post-transplant. Significant decrease in C-peptide by transplanted CD24+ (****p < 0.0001) and CD24+CD29+ (**p =0.001) SSCs, and a significant increase in plasma adiponectin by transplanted CD24+ (*p =0.0149) and CD24+CD29+ (*p = 0.0456) SSCs at 12 weeks. Significant decrease in C-peptide (**p =0.0005) by transplanted CD24+SSCs compared to CD24+CD29+SSCs. One-way ANOVA, Tukey’s multiple comparison test; mean ± S.D; results representative of 3 independent experiments, ns= non-significant. **l,** Imaging mass cytometry performed on femurs from 15-day old mice stained with a panel of heavy metal conjugated antibodies. **m,** A sample image defining the regions of interest (ROIs, dotted outlines) that were analyzed for distribution of SSC subsets. **n,** Spectral plots showing staining patterns for the mouse femur for various heavy metal conjugated antibodies. **o-r,** Representative plots showing the physical distribution of various annotated skeletal cell subsets within each ROIs as represented in (m). **s-v,** The calculated likelihood that each skeletal cell type is the nearest neighbor of a P1 cell (CD24+CD29+SSCs) within each ROI. **w,** Annotation (P1-P11) of skeletal cell subsets based on different markers.

**Figure 5:**
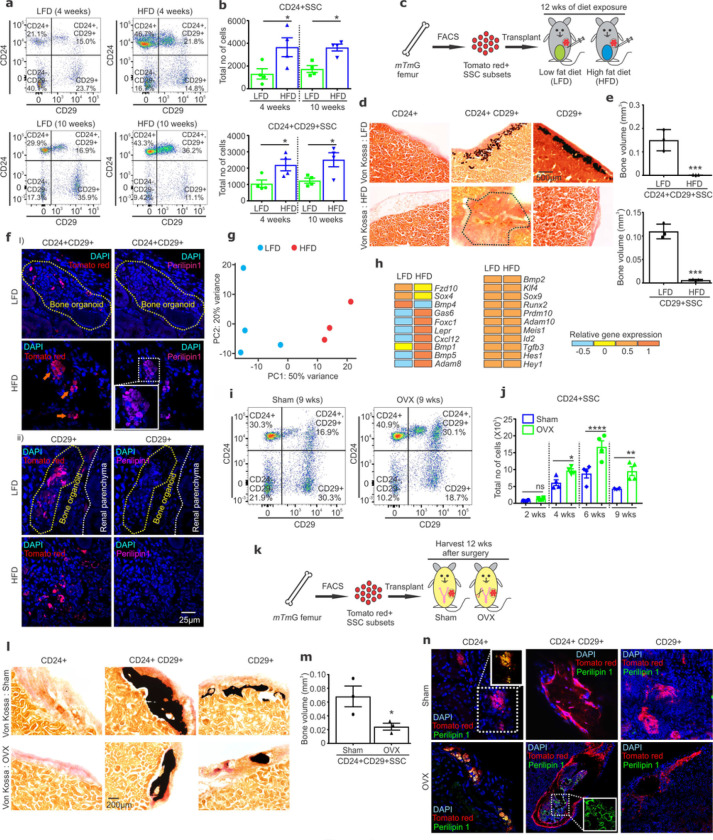
“Bone to fat” remodeling disorders reprogram lineage commitment by CD24+CD29+SSCs. **a,** Representative FACS plots showing the distribution of SSC subsets isolated from femurs of mice exposed to low fat diet (LFD, left) and high fat diet (HFD, right) for 4 (top) and 10 (bottom) weeks. **b,** Significant increases were seen in CD24+SSCs (*p = 0.0154 at 4 weeks; *p = 0.0476 at 10 weeks) (top), and CD24+CD29+SSCs (*p = 0.045 at 4 weeks; *p = 0.0257 at 10 weeks) (bottom) after HFD exposure. One-way ANOVA, Sidak’s multiple comparison test; mean ± SEM; n=4 mice/group. **c,**Sorted SSC subsets were transplanted into the kidney capsule of *MIP-GFP* mice exposed to LFD or HFD as indicated. **d,** Von Kossa staining (black) detects mineralized bone formed from transplanted CD24+ (left), CD24+CD29+ (middle) and CD29+ (right) SSCs implanted into *MIP-GFP* hosts exposed to LFD (top) and HFD (bottom) for 12 weeks. The dotted black line indicates the region with graft tissue. Scale bar 500μm. **e,** Quantification of bone volume when equal numbers of CD24+CD29+SSCs (top), and CD29+SSCs (bottom) were transplanted into mice exposed to LFD and HFD. CD24+CD29+SSCs (***p = 0.0047) and CD29+SSCs (***p =0.0003) display significantly reduced bone formation when exposed to HFD. Mean ± S.D; results representative of 3 independent experiments; 2 tailed Student’s t-test. **f,**Adipocyte differentiation by transplanted (tomato red) CD24+CD29+ **(i)**and CD29+ **(ii)** SSCs is confirmed by Perilipin 1 staining (magenta) in hosts exposed to LFD and HFD. DAPI (blue) for nuclei. Scale bar 25μm. **g,** Principal component analysis of RNA sequencing on FACS isolated CD24+CD29+SSCs from mouse femurs exposed to LFD or HFD for 4 weeks. **h,** Heatmap of differentially expressed genes in CD24+CD29+SSCs exposed to LFD and HFD. **i,** FACS plots showing distribution of SSCs isolated from the femurs of Sham (left) and OVX (right) mice 9 weeks after surgery. **j,** A significant increase in CD24+SSCs is observed in OVX mice 4 (*p = 0.049), 6 (***p < 0.0001) and 9 (**p = 0.0041) weeks after surgery. One-way ANOVA, Sidak’s multiple comparison test; mean ± SEM; n=4 mice/ group, ns= non-significant. **k,** Schematic representation of transplantation of sorted SSC subsets into kidney capsule of sham and ovariectomized (OVX) *MIP-GFP*hosts. **l,** Von Kossa staining (black) detects mineralized bone formed from CD24+ (left), CD24+CD29+ (middle) and CD29+ (right) SSCs transplanted into sham (top) and OVX (bottom) host mice 12 weeks after implantation. Scale bar 200μm. **m,** OVX causes a significant decrease (*p =0.041) in bone formation by transplanted CD24+CD29+ SSCs. Mean ± SEM; results representative of 3 independent experiment; 2 tailed Student’s t-test. **n,**Adipocyte generation from transplanted (tomato red) CD24+ (left), CD24+CD29+ (middle) and CD29+ (right) SSCs confirmed by Perilipin 1 staining (green) in Sham (top) and OVX (bottom) hosts. Enlarged view of dotted white boxes for Perilipin 1 staining. DAPI (blue) for nuclei.
